# Health Professionals’ Views on Key Enabling Factors and Barriers of National Multidisciplinary Team Meetings in Cancer Care: A Qualitative Study

**DOI:** 10.2147/JMDH.S240140

**Published:** 2020-02-14

**Authors:** Linn Rosell, Jessica Wihl, Mef Nilbert, Marlene Malmström

**Affiliations:** 1 Regional Cancer Centre South, Region Skåne, Lund, Sweden; 2 Lund University, Faculty of Medicine, Department of Clinical Sciences Lund, Lund, Sweden; 3 Department of Oncology and Hematology, Skåne University Hospital, Lund, Sweden; 4 Clinical Research Centre, Hvidovre Hospital and Copenhagen University, Copenhagen, Denmark; 5 Danish Cancer Society Research Centre, Copenhagen, Denmark; 6 The Institute for Palliative Care, Lund University and Region Skåne, Lund, Sweden; 7 Lund University, Faculty of Medicine, Department of Health Sciences, Lund, Sweden

**Keywords:** tumor board, rare cancer, healthcare team, treatment recommendation, decision-making, content analysis

## Abstract

**Purpose:**

Multidisciplinary team meetings (MDTMs) are an integral component of cancer care. Increasingly, virtual MDTMs are used to grant high-quality treatment recommendations across health-care regions, which expands and develops the local MDTM team to a regional or national expert network. We investigated health professionals’ experiences from national, virtual MDTMs for rare cancer with a focus on key enabling factors and barriers.

**Methods:**

Health professionals who participate in seven national, virtual MDTMs in Swedish health-care responded to a questionnaire exploring key enabling factors, barriers and opportunities for MDTM development. Conventional content analysis was used to identify thematic categories based on free-text responses.

**Results:**

Participants´ perspectives could be assigned into three categories ie, a national arena with potential for comprehensive knowledge and collaboration, prerequisites for decision-making and organization and responsibilities. These categories consisted of nine sub-categories that referred to, eg, collective competence, resources, clinical research, case discussion, meeting climate, patient-related information, MDTMs potential, referral and technical insufficiencies.

**Conclusion:**

National, virtual MDTMs represent a new multidisciplinary collaborative arena that introduces benefits as well as challenges. Consideration of key enabling factors and barriers may ease implementation and further optimize MDTMs in cancer care.

## Introduction

In cancer care, multidisciplinary team meetings (MDTMs) constitute a recurrent, weekly task for many health professionals and are recognized as a focal point of treatment recommendations. MDTMs contribute to coordinated care, improved quality of care and adherence to evidence-based guidelines.^1^ At the same time, MDTMs are resource-demanding with a growing number of case discussions and increasingly complex diagnostic paths and treatment options, which raises consideration of resource-effectiveness, possibilities to prioritize case discussions and risk of decision-making fatigue.^2^,^3^ These dual perspectives motivates evaluation of health professionals’ experiences from MDTMs.

### Centralized Treatment for Rare Cancers

Refined diagnostic procedures and novel treatment options, including development of personalized medicine programs, challenge health-care organizations to provide access to highly specialized skills across geographical regions. This is especially challenging for rare cancers, which are typically defined by an incidence of <6/100 000 persons per year and represent a heterogeneous group.^4^,^5^ Management of rare cancers is challenged by limited evidence for best practice, expert skills are typically confined to a few key health professionals and clinical research programs are hampered by the low incidence.^6^,^7^ As a group, rare cancers have reduced survival compared to other common cancers, are difficult to diagnose and require highly specialized knowledge and expertise for correct clinical management.^4^ Sweden has a population of 10 million, which implies that each rare cancer type develops in less than 600 individuals annually. To provide best possible services, grant sufficient expert knowledge and stimulate clinical development and research, treatment of certain rare cancers has been centralized to two-four national expert centers. These centers have established a national, virtual MDTM where newly diagnosed cases as well as all recurrences should be discussed. National, virtual MDTMs aim to grant treatment recommendation based on evidence or best possible expert opinion and to ensure equity of care across geographical regions, develop national expert networks and stimulate clinical research.

### Development of National, Virtual MDTMs

In Sweden MDTMs are held on local, regional and, more recently on, national level through video-based communication systems. To date, few studies have reported on implementation of national, virtual MDTMs, which makes the evidence-basis thin.^8^,^9^ Virtual MDTMs have been shown to connect geographically spread experts with benefits that particularly relate to improved coordination of care for patients in rural and remote areas and to treatment recommendations for complex cases and rare diseases.^2^,^10^–^14^ The MDTM network also provides possibilities for competence development for participating health professionals.^8^ Difficulties related to virtual MDTMs include dysfunctional technology, concerns about confidentiality, coordination challenges between hospitals and limited patient-centeredness.^6^,^9^,^10^

With the aim to develop and optimize national, virtual MDTMs, we investigated health professionals’ experiences of key enabling factors and barriers for national, virtual MDTMs for rare cancers.

## Materials and Methods

The study was designed as a descriptive, qualitative study with an explorative design. By using free-text answers form health professionals, key enabling factors, barriers and opportunities for development for national, virtual MDTMs were explored. The study is part of a larger research project aiming to address feasibility, function and health professionals experiences from national, virtual MDTMs in Swedish cancer care. Reporting are conducted according to the Standards for Reporting Qualitative Research (SRQR) guidelines.

### Context

In Sweden, treatment for seven types of rare cancers, has been centralized to national expert centers. This centralization was linked to establishment of national, virtual MDTMs to grant best possible treatment recommendation, develop national clinical networks, strengthen clinical research and improve patient care and outcome. Between 2015 and 2017, potentially curative treatments for penile cancer, anal cancer, vulvar cancer, gastroesophageal cancer, hepatobiliary cancer and cytoreductive surgery with hyperthermic intraperitoneal chemotherapy (HIPEC) were centralized. In 2017, a national, virtual MDTM was also initiated for childhood cancer with participation from the six regional pediatric oncology centers. Required participants in the various MDTMs are defined in the national standards of care and generally include surgeon, oncologist, pathologist, radiologist, contact nurse and MDTM coordinator. In Sweden, no formal MDTM training and/or evaluation is available.

### Respondents and Data Collection

Health professionals who regularly participate in the seven national MDTMs for rare cancers described above were eligible for the study. The research team developed a questionnaire based on an earlier study on health professionals’ experiences from local and regional MDTMs.^15^ Information about the study and a link to an online questionnaire was distributed to all participants (N=241) by e-mail. Two reminders were sent. Data were collected between 2017 and 2018. In total, responses were obtained from 125/241 (52%) invited health professionals. The scoring part of these data have been reported elsewhere,^8^ whereas the present study focuses on participants’ experiences based on 278 written free-text answers to the questions, “what’s your experience regarding profit/benefits of MDTMs? what is your experience regarding disadvantage/difficulties of MDTM? how would you wish to develop MDTMs?” Demographic data are presented in Table 1.

**Table 1:** Demographic Data

Gender		Profession		Discipline	
Women	45%	Physician	87%	Surgery	53%
Men	51%	Nurse	11%	Medicine/oncology	26%
No information	4%	Medical secretaries	2%	Radiology	6%
		None of the above	0%	Pathology	2%
				None of the above	14%

### Data Analysis

Free-text answers were analyzed with an inductive approach using conventional content analysis, which was motivated by limited availability of data from the study area.^16^ The answers were analyzed by three researchers (LR, JW and MM) with expertise in cancer care, qualitative methodology and MDTMs to grant different analytical perspectives. Initially, the text was read and re-read by all authors to get a sense of the whole to capture the concepts of the text. Thereafter, the analytical process was dynamic moving forward and back between the whole and the parts of the text. Notes were made through out the process. Words and meaning units were categorized in an initial coding scheme based on their relationship to create meaningful clusters.^16^ Similarities and differences in the initial coding were discussed until consensus was reached by all authors. The analysis resulted in three main categories (national arena with potential for comprehensive knowledge and collaboration, prerequisites for decision-making and organization and responsibilities). The categories were further split into nine subcategories (Table 2).

**Table 2:** Categories and Subcategories

Category 3.1	A National Arena with Potential for Comprehensive Knowledge and Collaboration	Category 3.2	Prerequisites for Decision-Making	Category 3.3	Organization and Responsibilities
3.1.1	Collective competence	3.2.1	Case discussion and adherence to treatment recommendation	3.3.1	Achieving the MDTMs full potential
3.1.2	Resource-demanding and suboptimal participation	3.2.2	Meeting climate	3.3.2	Referral to national MDTM
3.1.3	National arena for clinical research	3.2.3	Limited patient related information	3.3.3	Technical insufficiencies

### Ethical Approval

The participants’ confidentiality was granted by reporting the findings on group level. The study was ethically reviewed and granted permission by the Regional Ethics Review Board in Lund, Sweden (registration number 2016/195).

## Results

With a focus on key enabling factors and barriers for national, virtual MDTMs for rare cancers three main categories, ie, a national arena with potential for comprehensive knowledge and collaboration, prerequisites for decision-making and organization and responsibilities were defined (Table 2).

### A National Arena with Potential for Comprehensive Knowledge and Collaboration

#### Collective Competence

National MDTMs were described as an important and well-functioning arena for knowledge-sharing and for discussing complex cases in highly specialized diagnostic and therapeutic areas. Case discussion at national MDTMs were reported to contribute to enhanced individual competence and strengthening team competence. This was described by one respondent,

childhood cancer is a small specialty with great heterogeneity, that’s why it is invaluable to share knowledge and competence with colleagues outside our own clinic. [physician, medicine/oncology]

Through nation-wide referrals, participating health professionals are exposed to a considerably higher number of cases, which was described to contribute to increased experience. The educational perspective was regarded as advantageous and may be especially valuable for small-volume clinics, which is reflected in the quote

(national MDTMs) offers great educational opportunities because it is a small diagnose area, you can go through a whole professional life only seeing a handful. Here anyone interested can see all cases. [nurse, surgery]

The collective competences and experiences were described to contribute to a thorough discussion, to provide grounds for national consensus, contribute to adherence to standards of care and was regarded as a key enabling factor for decision-making. National MDTMs were also described to decrease the gap between experts in different geographical regions, which was perceived to be beneficial for collaborative professional networks.

#### Resource-Demanding and Suboptimal Participation

National MDTMs were perceived as important for high-quality treatment recommendations, but shortage of resources was described as a barrier to grant treatment within predefined lead times. The respondents reported suboptimal attendance, primarily related to lack of resources in radiology, pathology and oncology. Causes of suboptimal participation were reported to be irregular meeting dates and needs to coordinate participation with other health-care tasks. The MDTM was also perceived to be time-consuming and resource-demanding, particularly related to the preparatory work. Although active and well-prepared participants were described to grant effective case discussions, preparedness was reported to vary among the participants with negative influence on the quality of the case discussion. Some respondents also described lower commitment and participation in discussions of patients referred from other hospitals. To optimize participation the respondents suggested improvements including development of guidelines for mandatory attendance of key members with possibilities to invite specialists when relevant.

#### National Arena for Clinical Research

National MDTMs were described to have potential to increase clinical research collaborations and enable inclusion of patients in clinical trials. Several respondents, however, described a limited focus on clinical trials and it was suggested that designated time to discuss research protocols would enhance collaboration and stimulate research initiatives.

### Prerequisites for Decision-Making

#### Case Discussion and Adherence to Treatment Recommendation

National MDTMs were reported to be relevant and feasible fora for discussions of complex cases. Respondents reported confidence in access to national, multidisciplinary expertise in the decision-making process. A major aim of case discussion at MDTMs is to provide treatment recommendations according to national guidelines. However, lack of transparency in terms of compatible e-health system and privacy regulations were described as complicating factors. One respondent described that,

It is difficult to know what’s been documented when each clinic makes their own documentation, if you chose to oppose the conference (recommendation) you can avoid document it in the journal. [medical secretary, medicine/oncology]

Therefore, it was suggested that the coordinating national centre should be responsible for documentation to enhance transparency and that the MDTM teams should designate time for evaluation and feedback.

#### Meeting Climate

The respondents’ experiences of the case discussions varied greatly. Whereas some respondents described well-functioning meetings with structured discussions and an open meeting climate. Others described the meetings as sub-optimal with disorganized discussions, unresolved conflicts and stress related to needing to “perform” at a national arena. One respondent described,

we have a well-functioning local MDTM but at the national MDTM you feel the pressure to review right and work effectively. Before the conference I sometimes call our surgeon to coordinate and make an agreement on what we are going to present. [physician, radiology]

To enable optimal case discussions, the importance of an open meeting climate was emphasized, and some respondents reported a need for clarification of roles and responsibilities.

#### Limited Patient Related Information

National MDTMs for rare cancers were perceived to contribute to equity in care, increased patient safety and were also reported to provide an unofficial second opinion functionality. Though treatment recommendations from a national MDTM were considered important for the patient, some respondents expressed concerns about limited availability and consideration of patient-related information such as comorbidities, performance status, care needs and patients’ perspectives. Lack of relevant information was reported to lead to adjustments of and deviations from the recommendation given. One respondent described,

it is common that you don’t have any information about the patient before the conference, then you can’t make a correct judgment and contribute to the discussion. It’s sort of a hostage situation. [physician, surgery]

It was suggested that enhanced focus on patient-related information prior to the national MDTM would improve discussions quality. The respondents suggested that the referring physicians should participate to grant relevant recommendations and minimize needs for recurrent discussions.

### Organization and Responsibilities

#### Achieving the MDTMs Full Potential

Several respondents reported that the national MDTMs did not reach the full potential. Shortage of relevant resources, uncertainty of the assignment and a feeling of competition between participating treatment centres negatively influenced collaboration and lead to misunderstandings. One respondent reported,

To me the distribution of mandate is little unclear. We report a patient, present a short case history and then X (treatment center) decides what should be done. It´s not a discussion on equal terms, but maybe that’s the whole point. [physician, surgery]

In parallel, it was reported that implementation takes time. Better regional knowledge of the national MDTMs and agreements on meeting procedures were suggestion to ease implementation and collaboration.

#### Referral to National MDTM

According to the agreement on national MDTMs and the standards of care for the diagnoses in question, all patients within the areas defined should be referred to a national MDTMs. Respondents, however, reported sub-optimal compliance to the referral guidelines and described this as a potential barrier for patients’ access to equal health care. Reasons for not referring patients was motivated by obscure referral principles and prestige with hesitation having to ask a national MDTM for advice. Some respondents described case overload and argued for selection of complex cases. Transparent and accepted referral guidelines therefore likely represent a key success factor for national MDTMs.

#### Technical Insufficiencies

Participating hospitals used different technologies and e-health systems. Dysfunctional video-connections where participants could not see all participants or patient-related material were reported to lead to misunderstandings. Time-consuming, referral processes and complicated transfer of health-related information between centers, particularly within radiology, were also described as problematic. The respondents suggested better inter-operability of the e-health systems to increase effectiveness and encourage participation.

## Discussion

During the latest years, MDTMs have developed in some diagnostic areas from local team meetings to regional or national multidisciplinary networks. This study adds knowledge about health professionals’ experiences of key enabling factors and barriers for national, virtual MDTMs for rare cancers, which is essential for future improvements. Few studies today, have reported on implementation of national MDTMs^8^,^9^ and it is well known that a strategy for implementation is essential for the outcome.^17^ Damschroder et al (2009) established the theoretical framework, Consolidated Framework for Implementation Research (CFIR) consisting of five synergetic domains by which implementation is accomplished; the intervention, the inner and outer setting, individual characteristics and process. On a macro level, these domains can be used to explain and understand research findings and how implementation affect team’s performance.^18^ Therefore, we use the CFIR to discuss the main findings of this study focusing on key enabling factors such as strengthening professional networks (outer setting) as well as barriers such as suboptimal attendance (characteristics of individuals), resource constrains/lack of designated time (intervention) and uncertain assignments (intervention and inner setting).

In this study the respondents´ reported that national, virtual MDTMs provide support in decision-making, strengthen collaborations and professional networks, and develop individual and team-related competence, which is also supported by previous observations.^10^,^13^ The benefits of professional networks are supported by the CFIR domain outer setting which emphasize the importance of organizations promoting networking and teambuilding since this positively influence implementation by individuals sharing information and visions.^18^ Further, it has also been shown that competence -, and network development is particularly relevant for health professional in county hospitals or low volume centres,^8^,^19^ which is supported by this study.

As other developments in cancer care, the implementation of national, virtual MDTMs has not been without challenges. The results of this study indicate that the main challenges for national, virtual MDTMs is suboptimal attendance, time and resource constraints as well as experiences of unclear assignments and low adherence to referral guidelines. The results therefore suggest that the organizational change reached through the initiation of a national, virtual MDTM needs to be linked to behavioral change in participating health professionals. This is in line with the CFIR domain characteristics of individuals, which emphasizes that an organizational change begins with a change in individual behavior and that the degree to which new behavior are positively or negatively valued affect the willing to change.^18^ Therefore, promotional interventions can increase commitment and interactive participation.^19^,^20^

Several respondents reported resource constrains and lack of designated time to prepare for and participate in national, virtual MDTMs. These factors are interrelated, and lack of designated time has also in earlier studies been pointed out as a determinant for MDTM attendance.^1^,^12^,^19^–^22^ The CFIRs domain intervention includes adaption ie, to which degree the intervention can be adjusted to meet local needs, but also emphasize the importance of a balance between fulfilling the implementation and flexibility related to local needs,^18^ such as adaption to different working schedules. This emphasize the need of, at an early implementation state, clarify the value of participation in MDTM to motivate health professionals in investing the time and effort needed.^12^

MDTMs are resource-demanding, which motivates continuous work to ensure resource effectiveness. Initiatives to reduce caseload include mini-MDTMs for standard cases^19^,^20^,^23^ and selection of complex cases who benefits the most from full MDTM.^2^,^24^ Swedish guidelines for the seven rare cancers here studied call for referral of all newly diagnosed cases as well as all recurrences. The respondents claimed that all relevant cases are not referred, which may depend on several factors such as uncertainty of referral guidelines^23^ and recent implementation of national, virtual MDTMs.^14^ Hence, to improve MDTM effectiveness and meet the increasing demands on MDTM, structures for recurrent evaluations^19^ and transparent referral guidelines are relevant to develop.

The respondents described that uncertain assignments and responsibilities and suboptimal collaboration between hospitals prevented the national, virtual MDTMs from reaching their full potential. The CFIRs intervention domain indicate that complexity increases with the number of targets (in this case several participating hospitals and individuals) and relates to how the intervention affects the work processes.^18^ This suggests that it is important to clarify the national, virtual MDTM assignment^12^ and to ensure efficient communication about the service at an early state of implementation. This is supported by the CFIRs inner setting domain, which stresses the importance of well-functioning informal and formal communication and clarification of goals.^18^ In addition, leadership skills and use of rotating responsibility for chairing the meeting have been suggested to improve teamwork and to decrease conflict levels.^1^,^21^

In line with earlier studies, the respondents´ also reported suboptimal consideration of patient-related information, time constraints and non-attendance from core members^15^,^25^ as a barrier for relevant treatment recommendations. Although a relevant MDTM recommendation should be evidence-based and patient-centered,^21^ several studies show that the biomedical perspective dominate with less attention to other perspectives.^8^,^19^,^20^,^26^–^28^ Improvements in this field include structures for standardized documentation and presentation of patient-related perspectives.^19^

### Strengths and Limitations

Our study was conducted at an early state of implementation of national, virtual MDTMs and may therefore describe implementation challenges that have been resolved during the process. The study has limitations which needs to be considered when interpreting the results. These limitations include a response rate of 52%, no possibility for analysis of non-respondents, and that the free-text questions underlying the results of this study is not matched to the quantitative data in the core questionnaire. Further, the study is based on participants’ individual experiences and the results transferability is therefore difficult to value. The 125 responses, however, described various key enabling factors and barriers and the findings are supported by relevant research, which supports applicability in similar contexts.^29^ To ensure credibility the researchers moved forward and back between the original data and the analysis. Differences in interpretation between researchers was resolved through discussions until consensus was reached. The credibility is further strengthened by illustrating quotes in the text. Using a questionnaire, respondents representing different national, virtual MDTMs was reached which allows insights from several different perspectives.^29^

## Conclusions

This study is to our knowledge, the first to explore health professionals’ experiences of key enabling factors and barriers for national, virtual MDTMs for rare cancers. Consideration of the enabling factors and barriers herein identified may easy implementation and functionality of future MDTMs in cancer care.
